# Collectanea Medica: Consisting of Anecdotes, Facts, Extracts, Illustrations, &c.

**Published:** 1826-06

**Authors:** 


### COLLECTANEA MEDICA:

CONSISTING OF

### ANECDOTES, FACTS, EXTRACTS, ILLUSTRATIONS, &c.

*Relating to the History or the Art of Medicine, and the Collateral Sciences.*

---

Floriferus, ut apes, in saltibus omnia libant,
Omnia nos, itidem, depascimur aurea dicta.

---

ART. I.—*An Account of the present State of Medicine in Italy.* By Fr. W. OPPENHEIM, M.D. (From the *Edinburgh Medical and Surgical Journal*, Nos. lxxxvi. lxxxvii.)

THE above is the title of an article in a late Number of the Magazine of Foreign Literature, an excellent medical work, edited in Hamburgh by Dr. GERSON and Dr. JULIUS. It contains much interesting matter, from which we have made a selection, calculated to give a correct idea of the state of medical science in Italy, and to serve as a guide to those who visit that country for the purpose of adding to their stock of professional information.

Our author describes the institutions of the Italian States, according to the order in which he visited them, beginning with the kingdom of Sardinia, in which there are two universities, one at Genoa and one at Turin.

In the Genoese university, ten professors are employed in teaching the different branches of medicine and surgery. None of these professors, however, enjoy much celebrity; practical anatomy is shamefully neglected, and there is no anatomical museum. The languishing state of science in this university, is attributed by our author to its being under the direction of the Jesuits, who are in possession of its revenues, and expend considerable sums upon the purchase of theological books, while they almost entirely neglect, or give but little encouragement to, the cultivation of natural history, comparative anatomy, and the other departments of medical education. During the short-lived constitution of thirty days, the students ranked themselves on the side of the anti-royalists, in consequence of which the university was closed for three months, upon the return and restoration of the king.

The population of Genoa amounts to 90,000. It has two civil and one military hospital, besides a work-house.

The *Ospedale Pammatone* is very large, and externally resembles a palace more than a hospital. Its pillars, stairs, and balustrades, all of Carrara marble, lead the stranger to expect a commodious interior. But here he is miserably disappointed, and finds a total want of every thing suited to promote the health or comfort of the patients. The wards are very spacious, but badly lighted, imperfectly ventilated, and extremely filthy. The floors are made of tiles, half worn out, and scarcely ever cleaned. The bedsteads are of iron, have no curtains, and are ranged in three rows, two of which are so close to each other that they touch.

The patients are attended by nuns, belonging to the order of Nostra Donna del Rifugio. Three physicians and four surgeons are attached to this institution, which has 1600 beds, but is capable of containing 4000. When our author visited it in 1824, the number of patients amounted only to 826. Post-mortem examinations are here very rare, and the anatomical cabinet consequently very poor, containing only about a dozen dried preparations, and one skeleton! In 1821, the admissions amounted to 9344; the proportion of deaths to recoveries, as one to six.

*Spedale degli Incurabili,* is a handsome but badly situated building, containing 1000 beds, and destined for the reception of the aged, the poor, and the insane. Patients of the latter description occupy a separate wing of the building. Their wards are spacious, but here again we meet with three rows of beds; and our feelings revolt at the situation of these wretched beings, the greater number of whom are chained hand and foot to their iron bedsteads! Where such a mode of coercion is employed,—where the strait waistcoat is unknown,—where the physicians, Drs. Isola and Timoni, (we wish not to conceal their names,) hurry daily through this abode of misery, without giving a single direction to the nurse-tenders concerning the treatment of the patients, can we wonder that a cure is scarcely ever effected? We are sorry to find that, disgusting as such a scene must be, it is even surpassed in an asylum at Vienna, where Dr. Oppenheim has seen the lunatics not only chained, but caged, like beasts in a menagerie!

We feel particularly anxious that such a state of things should not exist unknown, and consequently unredressed. What Dr. Oppenheim has already disclosed to our professional brethren in Germany, we think it our duty to publish in Great Britain, in the hopes of thereby contributing to promote a reformation in the treatment of lunatics in Italy; the first step to a reformation being to attract public attention to the extent and nature of the abuse. While we feel it our duty to record this barbarous treatment of lunatics in one of the first cities in Italy, we are yet far from wishing to stigmatise the Italian character as inhumane, or their physicians in general as ignorant; for we ourselves recollect the existence, and that at no very distant period, of abuses not less enormous in Great Britain.

Dr. Oppenheim is doubtful whether to attribute to absolute want, or a degraded state of national character, the alms-begging which prevails in the Italian hospitals. "As I passed each bed, (he observes,) its sickly tenant stretched forth his meagre arms to implore for charity!"

*Albergo dei Poveri,* a splendid building, adorned with costly architectural ornaments, but deficient in more essential qualities. It contains 2000 persons, consisting of the poor, the aged, and many orphans. The internal management of this institution is much better than that of those already described. The paupers and orphans appeared clean, well clothed, and well fed.

In Genoa there are also a military hospital, containing 800 beds, and an institution for the instruction of the deaf and dumb. The latter was founded in 1801, by the Abbé Octavius Assarotti, who still presides over it, and pursues nearly the same mode of instruction as is usual in France. In Italy, as elsewhere, this malady has been observed to be much more frequent among the poor than among the rich. The state of this institution, which contains twenty boys and twelve girls, is highly creditable to the Abbé.

Turin, for a population of 80,000, has four civil hospitals. That of *S. Giovanni* is the largest, being capable of containing 600 patients; but, at the time of Dr. Oppenheim's visit, there were not more than 200 in it. The walls are large, and well ventilated. The proportion of deaths to recoveries, out of 4557 patients admitted in 1821, was as one to seven. There is a clinical ward in this hospital, containing twenty beds.

*Casa dei Pazzi* contained 260 lunatics, of whom more than one-third were chained. Their situation seemed even more deplorable than that of their fellow sufferers at Genoa. Although there are three physicians and three surgeons attached to this asylum, yet Dr. Oppenheim could not discover that any curative measures were ever employed.

The university of Turin has eleven medical and surgical professors. The number of medical students generally amounts to nearly 100. The medical practitioners of the kingdom of Sardinia are much divided as to the systems they pursue. The juniors who have studied in Paris adhere in general to the doctrines of Broussais. Some of the seniors are still Brunonians; and, what is singular, the younger Rasori has many fewer followers than might be expected.

The Grand Duchy of Tuscany has one university at Pisa, and three medical academies, viz. at Florenz, Pistoia, and Sienna. In order to obtain a license to practise, the student must not only have attended an hospital in one of the above towns for seven years, but must have practised during that period under the direction of the clinical professor, and must finally submit to an examination. Even those who have taken the regular academical degree of M.D. elsewhere, must attend one of the Tuscan hospitals for two years, before he is allowed to enter on private practice.

The academies at Pistoia and Sienna are too inconsiderable to claim attention. Our author therefore passes at once to the university of Pisa, which has nine medical and surgical professors. There is here, however, no school for teaching midwifery.

The university building is small and inconvenient, and contains a few lecture rooms, besides an anatomical and surgical theatre; but the latter is so badly lighted, that most of the operations are performed in the former.

The medical courses commence in November, and conclude in August. The students of medicine amount to about 200, of whom many are Greeks. Dr. Oppenheim had an opportunity of hearing one of the lectures on anatomy, and declares it was the worst he ever heard.

We shall give our author's account of the medical institutions at Pisa, in his own words:—

"*Ospedale Santa Chiara è Casa dè Trovatelli* contains 300 patients; the wards are clean, spacious, and lofty. The medical and surgical patients, in this as in the other Italian hospitals, are not separated from each other, but lie in the same ward. There is a clinical ward, with twelve beds, for the treatment of medical cases, under the direction of Professor Morelli; besides one for surgical cases, under the direction of Professor Vacca Berlinghieri. The attention of these professors is not, however, confined to the clinical patients, for they make remarks upon every interesting case in the hospital. The surgeon's visit is made at seven in the morning, the physician's at ten. The hospital pupils here, as in all the other Tuscan schools, wear a particular uniform, consisting of a red surtout and a white apron: as the domestics of the hospital are similarly dressed, they are scarcely distinguishable from the students.

"Professor Vacca Berlinghieri is extremely polite to strangers, and is very communicative upon professional subjects. I shall relate what I saw and heard while in his company. His method of lithotomy* is well known, from his three Memoirs on that subject. His success has undoubtedly been considerable; for, of twenty-nine patients upon whom he has operated, he has lost but two; and, of these, one was more than sixty-four years old. I saw two persons upon whom he had operated; one was a boy, four years old, who had been cut for the stone five days before. He seemed to be doing well, the greater part of his urine being already voided through the urethra, while the wound was beginning to heal. The other was a very melancholy case of a young man, on whom the operation had been performed fourteen weeks previously to my visit. The stone had been broken in the first attempt to extract it, and the fragments were successively removed, great care being taken by the Professor to leave no part of it in the bladder. He used injections, and every other usual precaution; but nevertheless, and although the wound had assumed an healthy appearance, the patient complained of calculous pains on the twelfth day, when the Professor discovered another piece of the stone in the bladder. In removing this he made no new incision, but merely dilated the original wound with his finger. This fresh irritation caused a violent inflammation of the parts, and the swollen part of the rectum, which had performed the office of a valve in preventing the fæces from entering the bladder, sloughed away, so that a fistulous opening was formed between the rectum and the bladder, and the urine was consequently evacuated partly per anum, and partly through a catheter introduced into the urethra. When the catheter was introduced only as far as the neck of the bladder, the urine flowing through it was mixed with fæcal matter; but, when it was pushed higher towards the fundus, the urine was natural. When I saw the patient, he was less hectic than he had been, but still the fistulous opening into the rectum remained; its calibre was, however, diminished.

"When this operation had been revived in France by Sanson, and improved in Italy by Vacca, who merely makes an incision into the neck of the bladder, it excited much attention, and was practised by many French and Italian surgeons with considerable success. Scarpa,

* He opens into the bladder from the rectum.

however, urged many objections against its safety, the most important of which was the danger of wounding the vesiculæ seminales. To this Vacca replied, by stating that, of eighty patients thus operated on, none had felt a diminution of their sexual powers. It is very remarkable that so celebrated a surgeon as Dupuytren should have so suddenly changed his opinion concerning the propriety of this operation. He now declares himself decidedly hostile to it, and yet, when I was in Paris in 1823, he was quite enthusiastic in its favour; for I heard him say, that the number of patients who suffer from a fistula after this operation, was not greater than that of those who died after any other!

"Calculous complaints are very rare in the neighbourhood of Pisa, and the majority of the cases operated on by Vacca come from Bologna, Genoa, and the Piedmontese countries, whose population subsist almost entirely on vegetable nutriment; a fact proving that the formation of stone does not depend upon a superabundance of nitrogen.

"Diseases of the large arteries are so unfrequent at Pisa, that there had been no operation there for aneurism during ten years."

Dr. Oppenheim, however, saw at Pisa one case of popliteal aneurism in a healthy man. It is singular that Vacca refused in this case to perform the operation; alleging as a reason, that he believed the patient's blood possessed too little *plasticity*,* and the arterial coats had too much inclination to suffer from distention or rupture, to authorise a reasonable hope of successful termination. He could not assign any intelligible grounds for this opinion, which was, however, justified by the event; for, another surgeon having performed the operation, hemorrhage took place on the third day, which was stopped by tying the femoral artery above the origin of the profunda. This resource proved also ineffectual; for a fresh hemorrhage occurred in three days after, and the patient died.

Dissection showed that all the coats of the artery had been divided by the ligatures, while no sufficient coagulum had been formed, and no attempt made towards the exudation of coagulable lymph.

What symptoms Vacca conceived to contra-indicate the operation in this case, we, as well as Dr. Oppenheim, are unable to guess.

"Fistula lachrymalis is a very frequent disease at Pavia, and Vacca has observed it to occur in women more frequently than in men, in the proportion of seven to one. His operation consists in making an opening into the sac, with a small straight bistoury; he afterwards widens the sac with a fine silver probe, and introduces into the duct a bit of extremely fine catgut, having a silk thread fastened to its upper extremity, by means of which it is secured above. In the course of a few days, the lower end of the catgut is forced through the nostrils by blowing the nose; it is then drawn down and detached from the silk thread, to which he fixes a small dossil of charpie, and is thus enabled to introduce the latter, from below upwards, into the lachrymal sac. The advantage of this method is, that the dossil being introduced into the sac through the lower opening, the superior external opening need not be enlarged or stretched so as to render it liable to inflammation.

* The probable meaning of this expression is, that the blood was deficient in coagulable lymph.

Vacca insists upon the necessity of dividing the tendon of the orbicularis muscle in this operation, as a portion of the sac lies directly under this muscle, and of course cannot be touched with the necessary escharotics; and, if this be not done, he contends that this portion of the sac will remain in a state of inflammation, and will occasion a relapse of the disease. In my opinion, the division of this tendon cannot be effected, without danger of also dividing the lachrymal duct.

"One of the most interesting cases I saw at Pisa, was an emphysema caused by fracture of the ribs on the right side of the thorax, together with injury of the pleura and lung, but without any external wound. The patient was brought to the hospital five days after the accident, when the emphysema was excessive, and had extended over the entire of neck, chest, abdomen, and scrotum. The patient felt very little pain, and was quite free from the usual symptoms, cough and expectoration of blood. He could lie on either side, but preferred lying on the uninjured side. His respiration was free, and he could make a deep inspiration without its causing uneasiness. The transverse fracture of the rib was quite evident on examination. This case is certainly extremely interesting, and seems inexplicable. Under other circumstances, it would have been necessary to make an immediate incision, but Vacca merely applied a few leeches, and the disease disappeared gradually.

"I saw here also a case of peculiar induration of the mammary glands, in a strong healthy woman, aged thirty, who had been but a few days in the hospital. Both breasts were equally affected, and were of a stony hardness; the indurated glands were movable, and the skin immediately covering them was very red, while in every other part of the breast it was quite natural as to colour. The temperature of the affected parts was elevated, and the nipples (as it were) pressed inwards. The woman said that the disease had commenced suddenly, and without any assignable cause, about seven years before, since which it had remained in the same state.

"In every other respect she felt herself remarkably well; did not feel any pain whatsoever in the breasts, and had no disorder of the digestive organs. There was no suspicion of syphilis, and the malady bore not the least resemblance to schirrus; for *both* of the mammary glands were attacked at the same time, their temperature was increased, and the skin red. This disease, too, had remained nearly stationary from the time of its first origin; and in its form the swelling did not at all resemble schirrus, being destitute of the knotty elevations and inequalities so peculiar to that affection. The axillary glands were quite healthy.

"Vacca treated this disease as a case of chronic inflammation. He commenced with a venesection, the woman's constitution being very strong; and he afterwards applied leeches to the part, and cloths moistened with aqua laurocerasi. I saw this treatment continued for three days, without effect.

"The operation of trepanning, which I never saw performed in France, and but once in England, during a very diligent attendance on the hospitals in both these countries, is by no means of rare occurrence

1

at Pavia. Neither are its results, on the whole, unfavourable. In two cases, a perforation was made through the mastoid process, for the purpose of giving exit to matter formed in its cells, in consequence of otitis.

"Vacca has lately abandoned an operation he was formerly in the habit of performing frequently; I mean tying the saphena vein in cases of varix and varicose ulcers. The success of this practice was for a time considerable, but cases afterwards occurred in which dangerous symptoms were occasioned, such as violent inflammation of the vein. In a few instances this inflammation proved fatal."

Dr. Oppenheim observes, that an operation occasionally attended with such dangerous consequences, ought never to be undertaken for the relief of a complaint in itself destitute of danger.

We subscribe most willingly to this opinion, having ourselves learned from an extensive experience the uncertain issue of tying the saphena vein. One case we shall not easily forget. A young person, otherwise enjoying perfect health, was admitted into an hospital in order to undergo this operation. It was performed. Inflammation of the venous system supervened, and the patient died in a few days. For further information on this subject, see Hodgson on Diseases of the Veins and Arteries; Mr. Carmichael on Varix and Venous Inflammation; Dublin Hospital Reports, vol. ii.; and an excellent article on *Varices*, in the Dictionnaire des Sciences Medicales.

*Florence.*—Population, 80,000.

There are two hospitals, besides a foundling hospital.

1. *Spedale de Santa Maria Nuova,* capable of containing 1200 patients, but Dr. Oppenheim found in it only 600 in April 1824. The wards are large and lofty. The lower wards are in the form of a cross, and contain from 150 to 200 beds. They are badly ventilated, and uncleanly. Six physicians and six surgeons are attached to this institution, and attend in rotation, each for one month. Dr. Oppenheim blames this practice, as subjecting the patients to a constant change of treatment. The clinical wards of this hospital contain fifty beds; and, what is remarkable, almost all the operations are performed by the pupils,—of course, however, in the presence of the professors. Dr. Oppenheim saw here many cases of compound fracture, which were treated according to Dupuytren's method. He saw a case of medullary sarcoma affecting the testicles: castration was performed, but in five days a new fungous growth began to arise from the wound.

"Another melancholy picture was presented, by a case of fungus of the antrum highmorianum, which had forced its way into the mouth. Indeed, I do not think I ever witnessed an hospital containing so many desperate diseases, such as hopeless cases of morbus coxarius, cancer, and abscesses, attended with confirmed hectic. Fistula lachrymalis is here also common; and I had an opportunity of seeing some cases of suppuration within the substance of the mastoid process, one of which proved fatal, from caries and effusion of matter in the dura mater. It was attended with symptoms of cerebral compression.

Scrofula in all its forms, such as tubercular phthisis, caries of the bones, &c. is not less frequent here than at Pisa. The Italians administer occasionally muriate of barytes in this affection, and Vacca often sends his scrofulous patients to the sea for the benefit of bathing.

The operation for cataract, employed both at Florence and Pisa, is depression or else reclination, the needle being introduced through the sclerotica.

2. *Ospedale de St. Bonaficio,* destined for the reception of lunatics, incurables, and those afflicted with cutaneous complaints, is capable of containing 1000 patients. The lunatics are here much better treated than at Pisa or Genoa: they are never chained, but are subjected to much milder modes of coercion, such as the strait waistcoat. Dark rooms, having the walls lined with padding to prevent the patients from injuring themselves, are used for the confinement of persons during the accession of the maniacal paroxysm. There is no care taken to provide amusements or employment for the patients, and, on the whole, their *moral treatment* is entirely neglected; so that Dr. O. justly remarks, it ought to be called an *asylum,* not an *hospital,* for lunatics, the latter name implying the application of proper curative means. The consequence of this neglect is, that a cure is scarcely ever heard of.

*Foundling Hospital.*—A well-managed institution, which receives annually from 1500 to 1800 infants. They are well taken care of, and remain one year in the house, after which they are sent to the country. The bedsteads are of iron, and each contains four separate divisions, in which are placed four children's beds. This arrangement facilitates the attendance of the nurses upon the children.

"The diseases observed among the foundlings are not numerous. Inflammation or blenorrhœa of the eyes is uncommon, a circumstance probably owing to the exclusion of a glaring light from the wards, and to their cleanliness and proper ventilation. Jaundice, and induration of the cellular membrane, are quite unknown here. A great number of children *are said* to fall a sacrifice to syphilis. When brought into the house, they are apparently healthy, but in the course of a few weeks, or even days, they become pale and thin, cry or rather *whimper* much, get an appearance of old age in the face, and often become covered, about the genitals, anus, &c. with pustules and small ulcers; they grow cachectic, and finally die in a state of marasmus. The exhibition of mercury in this affection is found to be quite useless. I myself concur in the opinion of Dr. Breschet, of Paris, who has observed a similar complaint among the infants in *l'Hospital des Enfans trouvés,* but does not conceive it to depend upon syphilitic taint. His observations render it probable that it arises from an insidious inflammation of the abdominal viscera; a view of the subject confirmed by the diminution in its mortality, since a mode of practice founded on this view has been adopted.

A small *lying-in hospital,* containing six beds, erected for the instruction of the Tuscan midwifes, is connected with the Foundling Hospital. In order to obtain a license to practise midwifery, the females must attend three courses of lectures on that subject; besides which, they must reside eighteen months in the institution. The anatomy of the female pelvis is taught by means of beautiful wax models, which can be taken to pieces.

Puerperal fever is rare at Florence. On an average, twins occur five times in the hundred. The cæsarean operation has been performed twice there, and in both cases was unsuccessful. The midwifery practice seems on the whole judicious, and the accoucheurs are not addicted to the unnecessary use of instruments in delivery. Professor Bigeschi speaks highly of the ergot, as a means of forwarding the progress of labour in tedious cases.

"I must not omit mentioning the celebrated Florentine wax-works, which exceed the Vienna collection, not only in number, but in execution and anatomical accuracy. What has been added lately is inferior to the old collection, especially in the colouring. I shall never forget my astonishment at seeing a representation of the distribution of the fifth pair of nerves. It left nothing to be wished for, and had every branch described by a Bock or a Meckel. In fact, on examining it, you could not determine which was the more to be admired,—the anatomist who made the dissection, or the artist who made the model. The late Professor Ucelli, who was not only an able anatomist, but an expert artist, enriched this collection with many beautiful specimens in comparative anatomy, well worthy of a minute examination. The imitations of plants, fruits, &c. are not less elegantly executed; but this part of the collection loses its value, from the circumstances that the objects represented are indigenous in Italy; and we do not find any imitations of rare or tropical plants.

"I did not observe so great a number of blind people in any Italian city as in Florence. Every good begging station in this city is occupied by a blind beggar, and those stations descend by hereditary right, from one generation to another. How great the profits of these beggars must be, appears from the answers of a young man, when asked how it happened that he could afford to marry:—"*Thank God I have a blind father; therefore, as long as he lives, I shall never want.*" In general, these blind beggars are attended by stout young men, so that the proper order of things is reversed; for he who can neither see nor work supports him who can do both!

"Here I cannot omit adverting to another custom, prevalent not only in Florence, but in the other Italian cities, and which must necessarily exercise an injurious influence on the state of the medical profession. The apothecary's shop is the physician's rendezvous; for his messages are left, not at his own house, but at the shop of the apothecary whom he patronises, or who patronises him.

"The Italians do not understand the comfort of the expression "at home," like us Germans, but spends all his leisure hours in the open air, in the street, and engaged in the "*dolce far niente.*" The first thing the Italian practitioner does in the morning, is to hurry to his apothecary's shop, for the purpose of learning what orders have been left for him. Meetings are held by physicians, and appointments made at the shop of the apothecary; and there the young physician, who is looking for practice, *must* loiter away his days: I say *must*, for, if he does not do so, he will not succeed. Every stranger, who is in want of a physician, sends for one to the apothecary; and every one, who has no family physician, does the same. The *understanding* relative to their mutual interest, which arises from this singular connexion between these branches of the profession, must prove injurious to the patient, at least so far as it increases the probability of his being made to swallow medicine, more with the view of increasing the bill, than of restoring his health. This custom evidently degrades the physician, by making him a sort of creature of the apothecary, and likewise occasions a most serious loss of time, just at that period of life when his time is most valuable."

*The Papal Dominion.*—There are two medical schools in the dominions of the Pope,—viz. one at Bologna, and one at Rome.

*Rome.*—The university at Rome is named *Della Sapienza,* and has fourteen medical professorships. This university has no museum whatsoever.

The medical clinic is in the *Ospedale di St. Spirito,* where practical anatomy is also taught. The surgical clinic is in the hospital at *St. Giacomo in Angustia.* There is no institution for the instruction of accoucheurs. The internal arrangement of the Roman hospitals is so peculiar, that it deserves particular notice. They are altogether ecclesiastical institutions, formed according to the notions of churchmen, and destined to serve rather as asylums for the administration of spiritual consolation, than for the cure of diseases. Accordingly, the physicians and surgeons are persons but of secondary importance in a Roman hospital, while the priests and confessors enjoy the chief authority! They alone are the resident officers; to them the admission of a new patient is first communicated; and they administer the first remedies, confession and the sacrament. Chance must decide upon the remaining part of the cure; for, after having taken care of the soul, they concern not themselves about the cure of the body! The hospitals are small, but on the whole rather clean. The bedsteads are generally made of iron, some with and some without curtains. Some of these hospitals are situated in the most unhealthy parts of the city. They are nine in number, and altogether contain about 2000 beds. In some there are separate wards for consumptive patients; for the opinion that consumption is contagious, is universal in Italy. In the lunatic asylum are 400 patients, on whom the whip and the chain are not spared! Some of the hospitals cannot be visited by strangers, except permission has been granted by the Pope, a favour of which his holiness seems to be very sparing. All the convalescents from the different hospitals are brought to that of the *Holy Trinity,* for the purpose of enjoying the benefit of a nutritious diet. Dr. Oppenheim finds fault with this arrangement; and we agree with him in thinking that the convalescents might be well fed in the different hospitals, without going to the trouble of removing them to this general convalescent hospital, where, after all, they are only permitted to remain three days!

The diseases of every nation are necessarily much influenced by the customs and domestic habits of the people, and the nature of the climate. He who has not been an eye-witness of it, cannot form any idea of the uncleanliness prevalent in the south of Italy. The stranger, on his first arrival at Rome, is amazed at seeing whole groups of people, "*gruppi dei otiosi,*" consisting of fathers, mothers, children, and friends of the family, all employed in performing for each other an office which we shall not name. Suffice it to say, they use their fingers for purposes elsewhere performed with combs! This custom is so general, that it has, as a matter of course, occupied the pencil of the artist; and, in the magnificent collection of pictures at Florence, is one in which Venus is seen thus elegantly employed on the head of Cupid! So much are the inhabitants accustomed to sleep two in one bed, that, when two strangers arrive at a country inn, and require two beds, their demand is considered unreasonable. The peasants of Rome and Naples look upon washing and cleansing the skin as quite unnecessary; and the upper ranks are not less negligent of the bath, than the ancient Romans were attached to its use. The great number of church holidays serves, indeed, in a certain degree, to keep the city from utter filth; for the streets through which the religious processions are to pass must be previously swept.

In so warm a climate, this utter neglect of cleanliness necessarily produces an abundance of cutaneous complaints; and accordingly the hospital *Della St. Maria,* containing 400 beds, is insufficient for the accommodation of patients so afflicted. Tinea capitis is treated in this hospital in the following singular manner:—The head is first smeared with butter, for the purpose of softening the scabs. When the scabs are removed, the head is shaved, and all the roots of the hair pulled out with a broad tweezers. The next step is to make forty or fifty incisions in the scalp with a razor. The free flow of blood from these incisions is favoured by making the child sneeze. The head is finally washed with cold water, and then rubbed with rancid oil. This *cutting* and *plucking* is repeated every four or five days, as fast as the hair begins to appear. This *cure,* which they commend for being *simple* and *radical,* lasts generally for six or eight months, and in obstinate cases for one or two years!

Vaccination is again much neglected under the present Pope, and of course small-pox is on the increase. There were about seventy small-pox patients in the hospital at the time of Dr. Oppenheim's visit.

We shall not enter into the sources of the "*aria cattiva,*" which renders Rome so unhealthy, but merely remark, for the benefit of such of our readers as may intend to visit that city, that its influence is most severely felt during the months of June, July, August, and September. The miasma produces, besides common agues, a very fatal species of fever, which is termed the malignant ague. "The patient becomes at once weary and weak, complaining of heaviness of his limbs, heat of skin, dull headache, and confusion of ideas, &c. The looks are wild, the face oftener pale than red; and, even when it is flushed, a yellowish white tinge is perceptible near the angles of the mouth. The belly is often tender to the touch, and the right hypochondrium swollen. The patient is sometimes costive, but not unfrequently diarrhœa is present from the beginning. Enough has been related to place it beyond doubt that this fever, at its commencement, is of a gastric character, and is attended with an inflammatory affection of the liver. After the above symptoms have continued for some time, the fit commences with a violent rigor, which is followed, in an inconceivably short space of time, with a general and excessive disturbance of the whole nervous system; picking the bed-clothes, subsultus tendinum, the most violent delirium, and a low muttering sort of raving, succeed each other rapidly, and without any apparent regularity. In short, the disease at one moment wears the aspect of a fever attended with excitement, and at the next has all the characters of typhus in its latter stages; and these two forms, alternating with each other, seem, if it were possible, combined in the same patient. After the fit, the patient feels a greater degree of depression than before. Vomiting often comes on at the height, or towards the end, of the paroxysm. The second fit commences five or six hours after the first; and the third begins after an intermission of about the same duration, unless, indeed, (which not unfrequently happens,) death has already closed the scene. *The third fit is always fatal!* Peruvian bark, exhibited in the largest possible doses, is the "*sacra ancora*" on which the Roman physicians place all their hopes. During the fit, blisters and sinapisms are applied to the extremities, while the head is assiduously cooled by means of cloths dipped in cold water. The instant the first fit has ceased, bark is given, and that with the greatest possible diligence, as they do not know the moment a second fit may commence. It is always a favourable symptom that the "patient bears the bark well, but it too often happens that the stomach immediately rejects it. When this is not the case, four, or even six, ounces of bark are exhibited in the course of the day. The second fit is then so much diminished in violence, that its accession causes but little disturbance, and the patient is saved. The progress of this disease is most rapid in strong, robust, and plethoric habits. Relapses also frequently occur. In 1825, the proportion of recoveries to deaths was as eighty-five to fifteen; in other years, it has been as eighty to twenty. All other remedies have proved ineffectual in this fatal disorder. Venesection, emetics, opium, &c. seemed only to hasten its fatal termination, so that the physicians now place no dependence upon any thing except bark, which is used in immense quantities at Rome. The quantity used in the *Ospedale St. Spirito* often amounts daily to forty or fifty pounds."

The latter statement of our author, we cannot help observing, accords ill with his previous sweeping censure, concerning the inattention prevalent in the Roman hospitals with regard to the *bodily complaints of the patients.* He only said the priests ~~[illegible]~~ concern not (v. p. 480.

Some successful trials had been made at Rome with the sulphate of quinine, both in this fatal and in the common forms of ague. When there are symptoms of a deranged state of the alimentary canal, the Romans place no reliance on emetics, but cleanse the primæ viæ with one drop of croton oil, previously to exhibiting the bark.

The following rules are laid down by the Roman physicians, for strangers who remain at Rome during the sickly season:—They must get up at six o'clock, and, having made a light breakfast, on biscuits, coffee, &c. they may go about their business until after eleven. At one o'clock they dine, and ought to sleep for a few hours after dinner. It is reckoned dangerous to be out at noon, sunrise, or sunset. Two hours after sunset, a walk is recommended; after which a slight supper may be taken. At twelve they ought to go to bed, and should sleep with but little bed-clothes. The windows of the bed-room should be kept shut during the night. Strangers are likewise recommended to indulge in cool drinks, and to be very abstemious with regard to wine, which ought to be diluted with water.

"Phthisis is not common at Rome, and its introduction has been attributed to the English! for it is universally believed to be contagious. When a consumptive patient dies, his clothes, furniture, and bed, are always burned. There exists, too, a Papal Bull, prohibiting the sale of such articles. Scrofula is not uncommon at Rome, and calculous complaints are of rather frequent occurrence. Professor Sisco, who has performed the operation of lithotomy on more than fifty patients, follows the method recommended by Cheselden,—i. e. the lateral operation: his success has been considerable, and he objects in strong language to the recto-vesical operation of Vacca. The Roman surgeons boast of great success in strangulated hernia. Sisco never tries to heal the wound by the first intention, because he considers the cure by suppuration as the only radical one, as it produces a complete solidification of the parts, and thus prevents the necessity of afterwards wearing a truss.

Aneurisms are not common in Rome; their cure is generally attempted according to Vansalva's method. The Roman surgeons *have hitherto never ventured to tie the artery in this disease, but always proceed at once to amputation when Vansalva's method fails!* and yet Rome is scarcely three days' journey from the residence of Scarpa!

In syphilis, mercury is now used both externally and internally. Buboes are never permitted to burst spontaneously; they are always opened with the lancet.

The doctrine of contra-stimulus has fewer advocates in Rome than in any other Italian city.

Bologna has 60,000 inhabitants; an university, two civil hospitals, an orphan-house, and a work-house. *Spedali della Vita* contains about 500 beds; the wards are spacious, well ventilated and clean. Professors Comelli and Tommasini superintend the medical clinic, and Professor Venturoli the surgical. It is unnecessary for me to detail the medical practice in vogue here, as it is generally known, and the doctrines of contra-stimulus are already sufficiently familiar to the medical world, through the medium of a journal published in Bologna, under the title "Giornali della nuova Dottrina Medica Italiana." Without discussing the merits or demerits of this doctrine, I may however remark, that the very large doses of medicines which its advocates are in the habit of exhibiting, have universally the effect of rendering the system so difficult to be acted on, that the doses must be constantly increased, in order to produce any effects. Thus, I saw a man under treatment for abdominal disease, on whose bowels half-a-drachm of jalap, and four ounces of castor-oil, had not the least effect, until their action was assisted by a purgative enema! We pass by the other hospitals, as affording nothing worthy of remark, except that their internal economy seems better regulated than that of other Italian hospitals.

The university reckons about 600 students. It is large and beautifully built, and contains not only a good museum of natural history, but a tolerable anatomical collection. The preparations with which it has been enriched by the present professor of anatomy, Mandini, are very fine; as are also those made by Professor Quadri, who formerly taught here, but now resides at Naples. In the wax-works, I was much struck by the beauty of the pieces representing the muscles: they are the works of Lelli and Madame Penarolini. The library contains about 150,000 volumes. The librarian, Professor Mezzofanti, is distinguished in the literary world by his uncommon talent for languages. The Botanical Garden is the best I saw in Italy.

*The Kingdom of Naples.*—A glance at the state of general literature in Naples, will best explain the state of medical science in that city. All foreign books, even those which have received the sanction of the Roman censors, are subjected to the revision of the Neapolitan censors. If, after a scrupulous examination, nothing is detected unfavourable to the king or church, the publication of the book is permitted, and a tax of four carolines on each volume must be paid by the publisher: this sum is exorbitant, when we consider the cheapness of Italian books. So heavy a tax is likewise imposed upon public prints, that it amounts to a prohibition of all foreign journals. These regulations render the advancement of science so slow in Naples, that what has long since become obsolete in other countries is there considered as a literary novelty. The Neapolitan physicians find it, therefore, impossible to keep up with the modern improvements in medicine, and consequently their practice is formed on antiquated models. Luckily for the inhabitants, the climate is so mild, that they seldom stand in need of medical aid, and may in general leave their complaints to the cure of the *vis medicatrix naturæ.* Little can be expected from an university in such a country, even though it boasts of such men as Vulpes, Quadri, and Lanza. The hospital accommodation, too, at Naples, is quite disproportioned to the number of the inhabitants, which amounts to 450,000.

Dr. Oppenheim then proceeds at some length to describe the *Lunatic Asylum* at Aversa, a small town, situated eight miles from Naples, on the road to Capua. This institution excels, indeed, all others in Italy, but still falls far short of similar institutions in England and Germany. The only peculiarity we think worth relating, arises from the habits and genius of the Neapolitan people. In English mad-houses, much reliance is placed in affording means of employing or amusing the convalescents. With this view, we provide the lower classes with the means of following their ordinary trades and occupations, while we offer to the rich the recreation of cultivating a garden, or reading in a library. At Aversa, what are the objects of amusement? Billiard tables, music, various petty national games, puppet-shows of all descriptions, and a variety of toys!

We would almost believe its inmates to be boys, not men: but men it seems they are, at least in Naples, where even the upper classes spend the whole day in the coffee-houses or at the theatre, while the lower orders visit the puppet-show at seven o'clock in the morning, after which they dance the "tarantala," and play the "morra." This systematic trifling and habitual idleness afford a sufficient excuse for the indignation of the priest described in Kelly's Memoirs, who, having dispersed a crowd of Lazaroni assembled around a puppet-show, held up the crucifix with which he had dealt his blows, and cried "*Ecco il vero Polcinello;*" an exclamation which at first appears ridiculous, if not impious, but really conveys a most degrading idea of the people whom he addressed.

The Lombard State forms at present a province of Austria, and the constitution of the two universities it possesses is modelled entirely according to the plan of that of Vienna. A certain course of study, which lasts for five years, is laid down, and no deviation from this plan is permitted. The disadvantages of a restrictive plan of study, which assimilates an university to a great school, are too obvious to require comment; and we cannot therefore help regretting, with Dr. O., that it should have been lately introduced into France. Our regret is increased by observing that this plan does not include a branch of medical study, to which we owe our chief progress in anatomy and physiology, and to whose aid we are to look for important additions to pathology,—we mean comparative anatomy. When (says Dr. O.) we find so important an omission, we must indulge in gloomy anticipations concerning the future progress of science in a state which was formerly so distinguished in comparative anatomy, and produced such men as Spallanzani, Valsava, Lancisi, &c.

Padua, a town with 20,000 inhabitants, has one spacious hospital capable of holding 300 patients. The wards are clean and well ventilated, and the beds are placed at a sufficient distance from each other. The clinical wards contain twenty-four beds. The medical department is superintended by Professor Brera; the surgical by Professor Ruggieri. The university reckons about 700 students. The building is very beautiful, and originally a palace, planned by Palladio. The cabinet of natural history is tolerable. Fabricius ab Aquapendente, Prosper Alpinus, Morgagni, and other celebrated men, once ornamented this school. Brera, the present professor of the practice of physic, is a zealous advocate of the system of contra-stimulus, and of course propagates this doctrine among his pupils. It has, however, found fewer advocates among the private practitioners at Padua, than among those at Bologna.

Pavia has 22,000 inhabitants. The town hospital is well situated and very roomy, and contains 400 beds. It is better calculated for the purposes of an hospital than any other institution in Italy, having been originally built for the accommodation of the sick, and not, like the rest, for a monastery or a palace. There are four clinical wards, viz. one for medicine, one for surgery, one for diseases of the eye, and one

(as the catalogue has it) "*per la istruzione di maestri in chirurgia e flebotomia.*" Connected with the hospital is a small institution for lying-in women.

The university is attended by about 400 students. The building is large, and the architecture fine. No university in Italy can boast of such rich cabinets of natural philosophy, chemistry, natural history, and anatomy. The latter, commenced by Rezia, received many valuable additions from the celebrated Scarpa, lately a professor at Pavia. Burserius, Tissot, and the two Franks, formerly taught at Pavia.

---

